# MicroRNA-275 targets sarco/endoplasmic reticulum Ca^2+^ adenosine triphosphatase (SERCA) to control key functions in the mosquito gut

**DOI:** 10.1371/journal.pgen.1006943

**Published:** 2017-08-07

**Authors:** Bo Zhao, Keira J. Lucas, Tusar T. Saha, Jisu Ha, Lin Ling, Vladimir A. Kokoza, Sourav Roy, Alexander S. Raikhel

**Affiliations:** 1 Department of Entomology and Institute for Integrative Genome Biology, University of California Riverside, Riverside, California, United States of America; 2 Graduate Program in Genetics, Genomics and Bioinformatics, University of California Riverside, Riverside, California, United States of America; University of Cambridge, UNITED KINGDOM

## Abstract

The yellow fever mosquito *Aedes aegypti* is the major vector of arboviruses, causing numerous devastating human diseases, such as dengue and yellow fevers, Chikungunya and Zika. Female mosquitoes need vertebrate blood for egg development, and repeated cycles of blood feeding are tightly linked to pathogen transmission. The mosquito’s posterior midgut (gut) is involved in blood digestion and also serves as an entry point for pathogens. Thus, the mosquito gut is an important tissue to investigate. The miRNA aae-miR-275 (miR-275) has been shown to be required for normal blood digestion in the female mosquito; however, the mechanism of its action has remained unknown. Here, we demonstrate that miR-275 directly targets and positively regulates *sarco/endoplasmic reticulum Ca*^*2+*^ *adenosine triphosphatase*, which is implicated in active transport of Ca^2+^ from the cytosol to the sarco/endoplasmic reticulum. We utilized a combination of the gut-specific yeast transcription activator protein Gal4/upstream activating sequence (Gal4/UAS) system and miRNA Tough Decoy technology to deplete the endogenous level of miR-275 in guts of transgenic mosquitoes. This gut-specific reduction of miR-275 post blood meal decreased *SERCA* mRNA and protein levels of the digestive enzyme late trypsin. It also resulted in a significant reduction of gut microbiota. Moreover, the decrease of miR-275 and *SERCA* correlated with defects in the Notch signaling pathway and assembly of the gut actin cytoskeleton. The adverse phenotypes caused by miR-275 silencing were rescued by injections of miR-275 mimic. Thus, we have discovered that miR-275 directly targets *SERCA*, and the maintenance of its level is critical for multiple gut functions in mosquitoes.

## Author summary

Female mosquitoes transmit numerous devastating human diseases. The mosquito gut, in addition to its primary function as a site of blood digestion, represents the entry point for pathogen colonization in mosquito vectors. The conserved microRNA, miR-275, was shown to be required for blood digestion and egg development. In this study, we investigated the target of miR-275 contributing to the regulation of mosquito gut functions. We achieved spatiotemporal suppression of miR-275 using a transgenic Tough Decoy RNA approach in the *A*. *aegypti* female mosquito gut. Furthermore, we have uncovered that miR-275 targets *sarco/endoplasmic reticulum Ca*^*2+*^*- adenosine triphosphatase (SERCA)*, affecting numerous gut functions including blood digestion, production of digestive proteases, and assembly of the gut actin cytoskeleton. SERCA is essential for maintenance of Ca^2+^ homeostasis, and its disturbance, in humans, leads to cardiac hypertrophy, heart failure and cancers. Therefore, the finding that the miRNA miR-275 targets SERCA not only contributes to the knowledge of mosquito gut regulation but also significantly adds to the general understanding of mechanisms governing this critical molecule.

## Introduction

Female hematophagous mosquitoes require vertebrate blood to support rapid egg development. While feeding on an infected individual, female mosquitoes acquire pathogens, which are transmitted in subsequent blood feedings. The mosquito gut represents the entry point for pathogens, and it is here that essential pathogen life stages occur for further pathogen dissemination in the mosquito organism and eventual transmission. Therefore, understanding the regulatory mechanisms governing gut functioning in the process of blood utilization is critical for elucidating interactions between pathogens and their mosquito hosts. The mosquito *Aedes aegypti* is the major vector of arboviruses such as Dengue and yellow fever, chikungunya and Zika, and thus is considered an important insect for vector biology research.

Recent studies have implicated blood feeding in initiating global changes in the mosquito transcriptome; however, these changes are not limited to protein-coding mRNAs, but also to a large number of non-coding RNAs [1, 2]. MicroRNAs (miRNAs) are small non-coding RNA molecules of ~21 nucleotides in length that play significant roles in post-transcriptional regulation by forming hybrids with sequences located in the coding region or 3’ UTRs of target mRNAs [3]. The general outcomes of miRNA-mRNA interaction are post-transcriptional repression by degradation of mRNAs and/or translational repression [4]. Furthermore, findings indicate that miRNAs have the ability to stabilize mRNA or activate translation [5–7]. Recent studies have revealed that miRNAs play an important role in diverse biological functions in mosquitoes, such as blood digestion, reproduction, *Plasmodium* invasion, viral immunity and *Wolbachia* infection [8].

As various miRNA expression patterns are being uncovered, researchers encounter a rising challenge in discovering miRNA contributions to numerous physiological, developmental and other biological processes. Although genomic and bioinformatics methods accelerate identification of new miRNAs, limitation of available technologies for *in vivo* study has restricted exploration of miRNA functions in mosquitoes. In recent years, multiple tools to study miRNA functions *in vivo* have been developed. The approaches currently used for loss-of-function analysis fall into two categories, each with their own caveats. Silencing of mature miRNAs by chemically modified synthetic oligonucleotides (for example, antagomir) has a limitation for spatial information. The miRNA Tough Decoy (TuD) approach is based on expression of short transcripts with hairpin structure containing multiple binding sites for a specific miRNA of interest [9, 10]. Meanwhile, the Gal4/UAS system could provide robust spatiotemporal specific transgene expression [11]. A combination of these two approaches represents a versatile tool for achieving spatial and temporal analysis and uncovering miRNA functions. We applied this Gal4/UAS-TuD approach to study spatiotemporal actions of miR-275 in the gut of female *A*. *aegypti* mosquitoes.

Previously, Bryant et al. used specific antagomir depletion of the conserved miR-275 to show that it is required for blood digestion and egg development [12]. However, gene targets contributing to these phenotypes have not been identified. In the present study, we have achieved spatiotemporal suppression of miR-275 using the Gal4/UAS-TuD approach in the female *A*. *aegypti* mosquito gut. In doing so, we have uncovered that miR-275 directly targets the *sarco/endoplasmic reticulum Ca*^*2+*^ *adenosine triphosphatase* (*SERCA*) gene, the mRNA level of which is diminished in the background of silenced miR-275. In the mosquito gut, miR-275 and its target *SERCA* are essential for controlling digestive enzyme production and maintenance of microbiota. Furthermore, our evidence suggests that SERCA and Notch signaling control the gut actin cytoskeleton, which is disrupted in the background of silenced miR-275. SERCA is involved in active transport of Ca^2+^ from the cytosol to the sarco/endoplasmic reticulum stores, thereby maintaining Ca^2+^ homeostasis. SERCA disruption leads to multiple abnormalities, including cardiac hypertrophy and heart failure in humans and *Drosophila*, and cancers in humans [13–15]. Therefore, in addition to its importance in controlling gut functions in the mosquito, the discovery of the miRNA-275 that directly targets *SERCA* represents an essential step in our understating of its regulation in general.

## Results

### The spatiotemporal silencing of miR-275 in the gut using the Gal4/UAS-TuD approach

miR-275 is highly induced in the *A*. *aegypti* gut following a blood meal (post blood meal, PBM), suggesting its importance for blood digestion [16]. To determine the gut-specific roles of miR-275, we spatiotemporally silenced this miRNA by means of the TuD in combination with the previously established gut-specific *Aedes carboxypeptidase* (CP) promoter-linked Gal4/UAS system (S1A Fig) [17]. The *piggyBac* transposable element was used to generate the UAS-miR-275-TuD transgenic lines. The transgenic cassette was assembled with a dsRed marker gene driven by the eye-specific *3×P3* promoter (S1B Fig). The UAS-miR-275-TuD construct contained the UAS linked to a miR-275-TuD sequence, followed by a SV40 polyadenylation signal (S1B, S1C and S2 Figs).

Embryos of *A*. *aegypti* were injected with a mixture of the UAS-miR-275-TuD construct and a helper plasmid. A total of about 1000 embryos were injected, with 208 G_0_ mosquitoes surviving to adulthood (127 males and 81 females). G_0_ males were out-crossed individually with 5 wt females, forming 127 male families (M1-M127). G_0_ females were out-crossed with one wt male giving rise to 81 female families (F1-F81). The dsRed marker was used to screen positive G_1_ progeny (larvae and pupae), and two individuals (from F1 and F17 female family, respectively) were selected based on the presence of strong eye-specific dsRed expression. The progeny of these two individuals were multiplied and established as homozygous UAS-miR-275-TuD responder lines (named as F1 line and F17 line). CP-Gal4>UAS-miR-275-TuD hybrid mosquitoes (16-1/F1 line and 16-1/F17 line) were produced by crossing the homozygous CP-Gal4 driver line (16–1 line) with each homozygous UAS-miR-275-TuD responder line. CP-Gal4>UAS-miR-275-TuD hybrid mosquitoes were selected based on the coincident presence of both EGFP and dsRed eye-specific markers (S3A–S3D Fig). Genomic PCR analysis confirmed the presence of both CP-Gal4 and UAS-miR-275-TuD transgenes in hybrid lines (S3E Fig).

**Fig 1:**
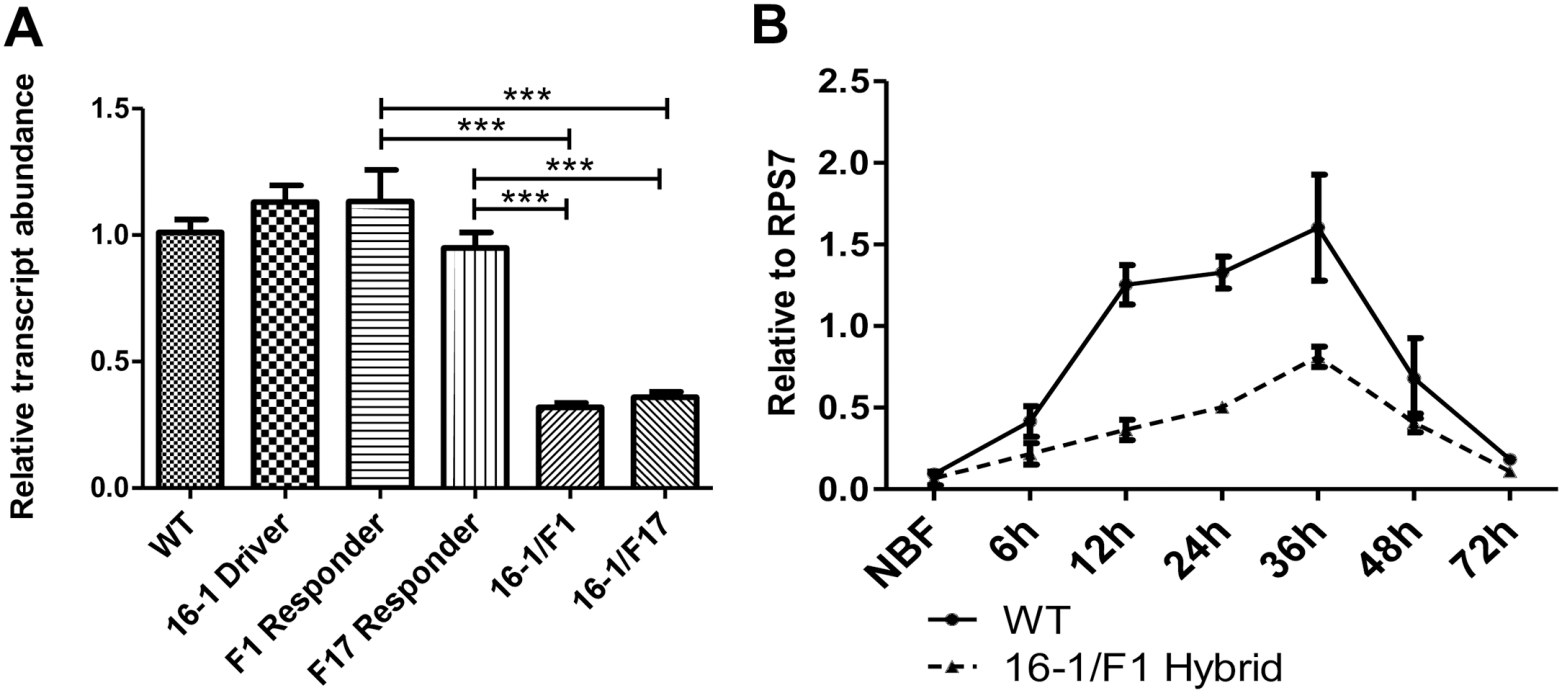
miR-275-TuD reduces miR-275 levels. (A) When compared with controls (wt, 16–1 Driver, F1 Responder and F17 Responder), miR-275 levels in guts at 24h PBM are lower in CP-Gal4>UAS–miR-275-TuD (16-1/F1, 16-1/F17) female mosquitoes. (B) Gut-specific depletion of miR-275 in CP-Gal4>UAS–miR-275-TuD female mosquitoes compared with wt. Samples were collected from non-blood fed mosquito guts (NBF) and at 6, 12, 24, 36, 48, and 72h PBM.

To assess whether miR-275 was effectively depleted in CP-Gal4>UAS-miR-275-TuD hybrid mosquitoes, quantitative reverse-transcriptase PCR (qRT-PCR) was performed to analyze levels of this miRNA in the gut. Compared with control mosquitoes (wt, 16–1 driver line and two responder lines), miR-275 levels were significantly lower in both CP-Gal4>UAS-miR-275-TuD hybrid lines 24 hours PBM, demonstrating efficiency of the miR-275-TuD RNA (Fig 1A). The level of miR-275 was unaffected in the fat bodies of these transgenic mosquitoes when compared with wt controls, demonstrating tissue specificity of the depletion (S3F Fig). We also performed qRT-PCR analysis to monitor mature miR-275 levels at several time points PBM during the first reproductive cycle. Total RNA samples from female mosquito guts of wt and transgenic hybrid mosquitoes were prepared at non-blood-fed stage (NBF) and at 6, 12, 24, 36, 48 and 72 h PBM. In both wt and transgenic line, miR-275 level was induced after a blood meal, reached its peak by 36h PBM and declined thereafter. However, the level miR-275 was dramatically lower in guts of transgenic mosquitoes than those of wt at the same time points (Fig 1B). Together, these results suggest that the TuD method specifically decreases miR-275 level in guts.

### Blood meal digestion defects caused by miR-275 silencing

**Fig 2:**
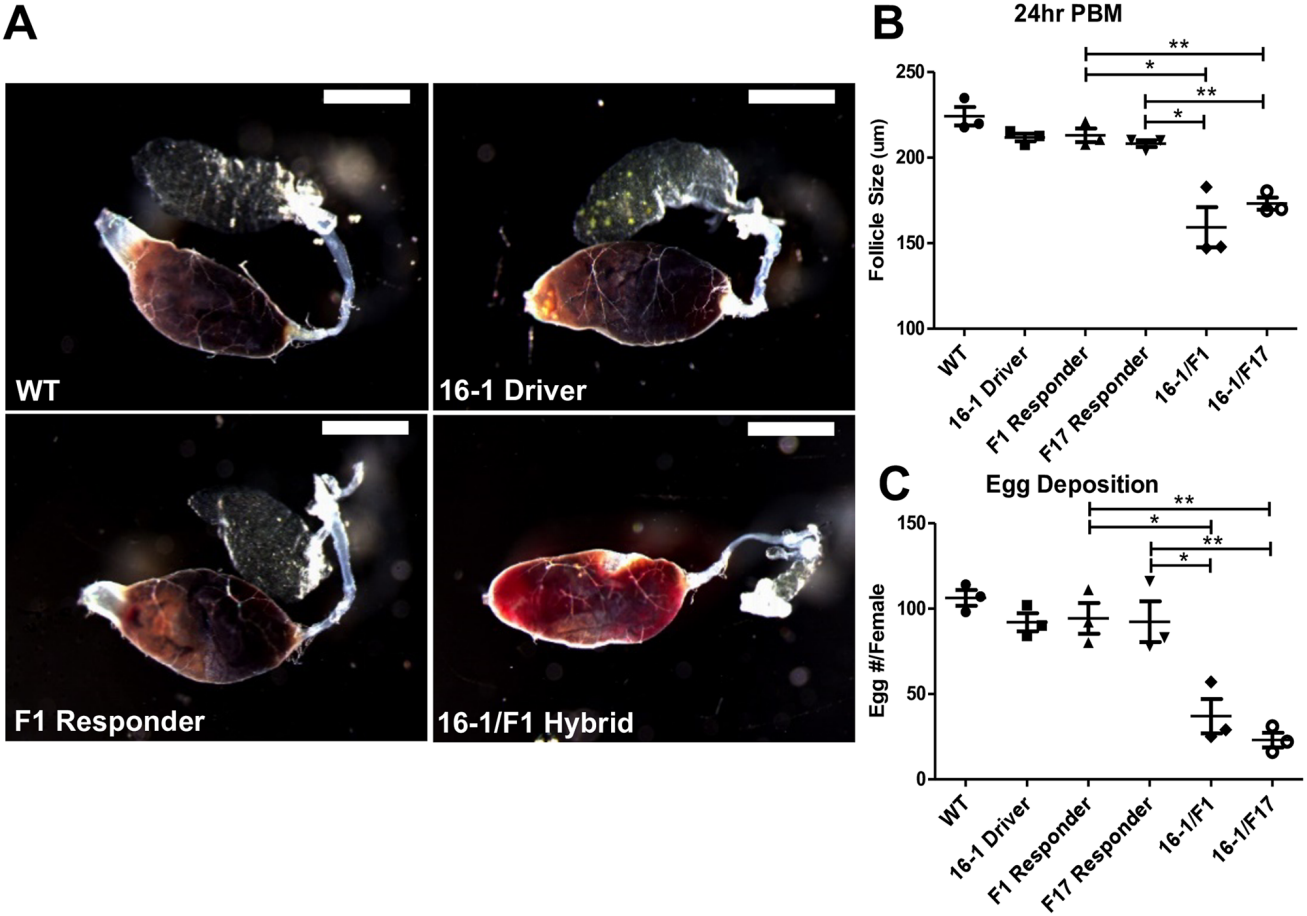
Effect of gut-specific depletion of miR-275 in blood-fed *A*. *aegypti* female mosquitoes. These lines include wt, 16–1 Driver, F1 Responder, F17 Responder and CP-Gal4>UAS–miR-275-TuD lines (16-1/F1, 16-1/F17). (A) Female mosquito guts at 24 h PBM. Images of guts were captured from under the Leica M165FC stereomicroscope (scale bar for guts: 1 mm). (B) Average follicle size of CP-Gal4>UAS–miR-275-TuD female mosquitoes compared with controls at 24 h PBM. (C) Average egg numbers per female mosquito (Egg #/Female) for different mosquito lines. Values represent average ±s.e.m. from three combined biological replicates; *P < 0.05; **P < 0.01.

**Fig 3:**
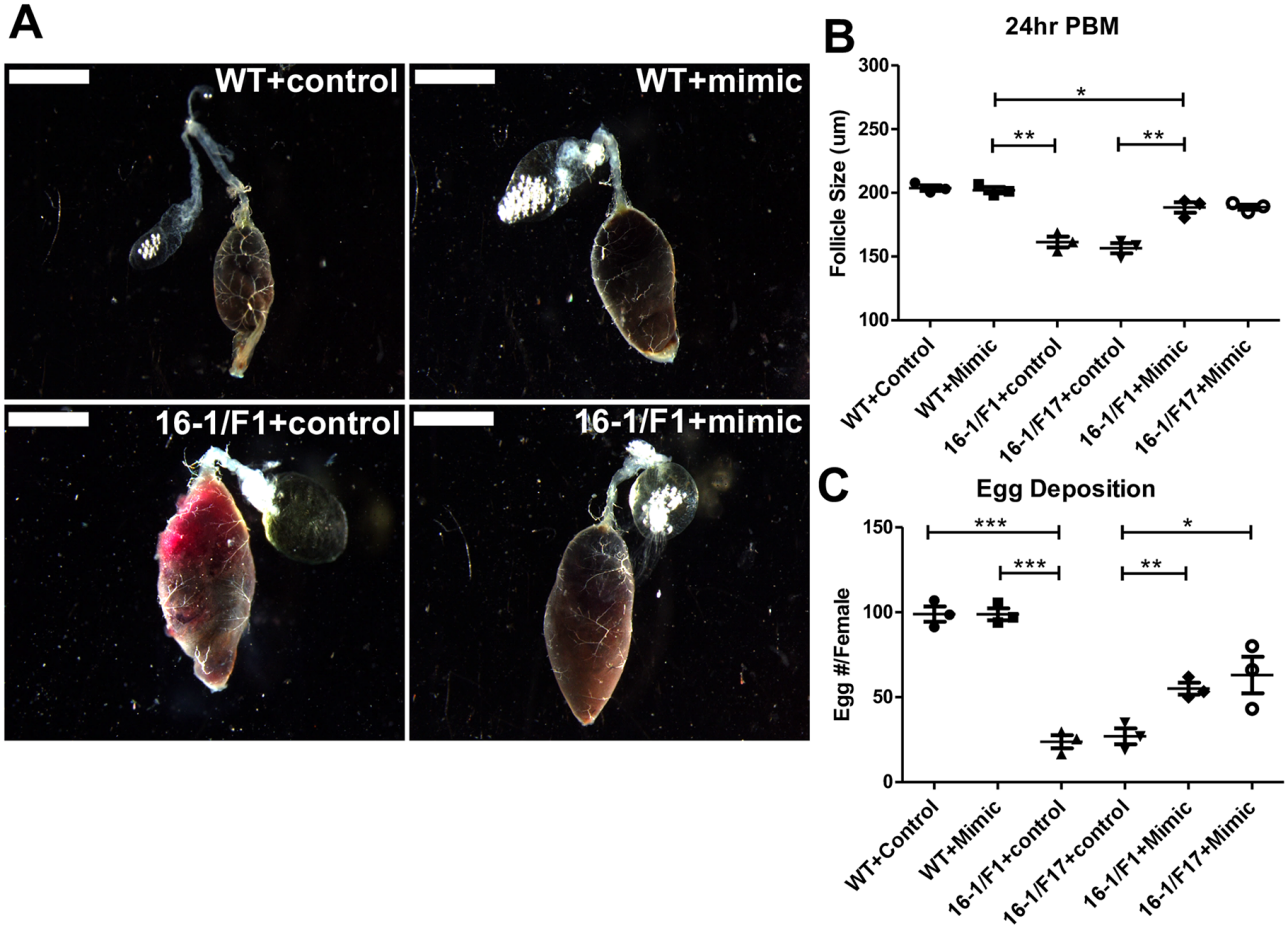
miR-275 mimic rescues the phenotypes in blood-fed CP-Gal4>UAS–miR-275-TuD female mosquitoes. These lines include wt, 16–1 Driver, F1 Responder, F17 Responder and CP-Gal4>UAS–miR-275-TuD lines (16-1/F1, 16-1/F17). Female mosquito guts were sampled at 24 h PBM. (A) miR-275 mimic rescued the phenotype of abnormal blood digestion in CP-Gal4>UAS–miR-275-TuD (16-1/F1) female mosquitoes (Scale bar for guts: 1 mm). (B) miR-275 mimic rescued the phenotype of reduced follicle size. (C) miR-275 mimic rescued the phenotype of aberrant egg number. Values represent average ±s.e.m. from three combined biological replicates; *P < 0.05; **P < 0.01; ***P < 0.001.

Blood remained partially undigested (light red bolus) in guts of the CP-Gal4>UAS-miR-275-TuD females at 24h PBM; at the same time point, normal digestion (dark brown bolus) was observed in the wt mosquitoes, as well as in the driver and two responder lines (Fig 2A, S6 Fig). Although miR-275 silencing was gut-specific, we observed defects in egg development likely linked to abnormal blood digestion. In wt, driver and responder lines, the average size of primary follicles was 205–225 μm in length 24h PBM, with very small nurse cells at the apex of the follicle. However, in both hybrids, the length of primary follicles were 145–185 μm on average, and nurse cells were substantially larger than controls indicating a delay in development of these follicles (Fig 2B, S4A and S6 Figs). The number of eggs laid by hybrid females was drastically reduced to 23–37 eggs/female, in comparison with the 92–106 eggs/female by controls (Fig 2C, S4B and S6 Figs). We then tested whether these adverse phenotypes could be rescued by miR-275 mimic. Indeed, partial rescue of the above-mentioned phenotypes was observed after the mimic injection. Blood digestion, gut structure, follicle size, egg shape and egg number per female were partially restored (Fig 3A–3C, S5A, S5B and S6 Figs).

### miR-275 targets *SERCA*

Next, we used a computational approach to predict putative target gene(s) for miR-275. Five miRNA target prediction programs—TargetScan, PITA, miRanda, RNA hybrid and a program developed in-house—were used to identify potential targets of miR-275 in *A*. *aegypti*, as previously described [18]. Predicted targets between each of the five programs were assessed and only one gene—*SERCA*—was detected by all five programs (S1 Table).

**Fig 4:**
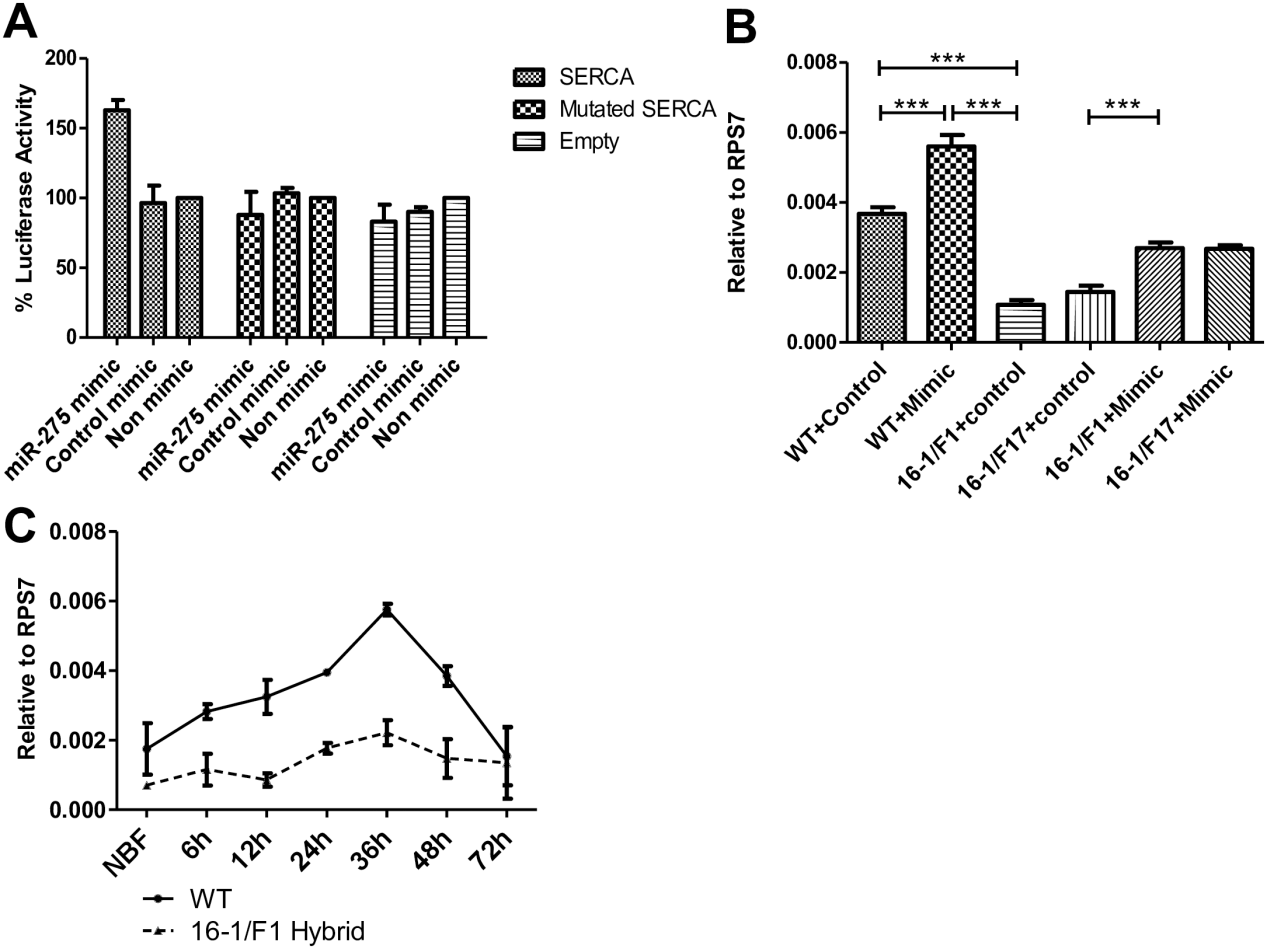
miR-275 targets *SERCA* gene. (A) Dual luciferase reporter assay for SERCA. Three reporter constructs, psiCHECK-2-SERCA (SERCA), psiCHECK-2-ΔSERCA (Mutated SERCA), and psiCHECK-2 (Empty) were co-transfected with miR-275 mimic, control miRNA (control mimic) or Drosophila Schneider S2 cell culture medium (Non mimic). Values represent the percentage activity (Δ fold activity×100). Δ Fold activity of psiCHECK-2 (Empty) and no-mimic treatment is set as 1. Percentages are shown as the average ±s.e.m. from three combined biological replicates. (B) miR-275 mimic elevates SERCA transcript levels in wt and CP-Gal4>UAS–miR-275-TuD (16-1/F1, 16-1/F17) female mosquitoes. Values represent average ±s.e.m. from three combined biological replicates; ***P < 0.001. (C) SERCA transcript levels decreased in CP-Gal4>UAS–miR-275-TuD female mosquito guts.

To validate the miR-275 target, we assessed the *SERCA* 3'-UTR response to miR-275 *in vitro*. The *SERCA* 3’-UTR that includes a predicted miR-275 binding site was cloned into the psiCHECK-2 vector downstream from the *Renilla luciferase* reporter gene, resulting in the psiCHECK-2-SERCA construct (S7A Fig). In addition, the construct containing a mutated miR-275 binding site within the *SERCA* 3’UTR was also produced, yielding a psiCHECK-2-ΔSERCA construct (S7B Fig). The empty psiCHECK-2 vector served as another control construct. Luciferase activity significantly increased in *Drosophila* S2 cells co-transfected with psiCHECK-2-SERCA and miR-275 mimic (Fig 4A). No changes in luciferase activity were observed in cells transfected with the mutated miR-275 binding site (psiCHECK-2-ΔSERCA) or the negative control vector (psiCHECK-2) in response to the miR-275 mimic (Fig 4A).

To further confirm *SERCA* as a miR-275 target *in vivo*, we measured *SERCA* transcript levels in the guts of miR-275-depleted female mosquitoes and observed significant decreases in *SERCA* mRNA when compared with wt mosquitoes (Fig 4B). We also performed qRT-PCR analysis to monitor *SERCA* transcript levels at several time points PBM during the first reproductive cycle as described above for mature miR-275. In wt, the level of *SERCA* transcript was elevated after a blood meal, reached its peak by 36h PBM and declined thereafter, corresponding to the level changes of miR-275 in the gut at the same time points (Fig 4C). It is possible that a supplement of exogenous miR-275 mimic could rescue *SERCA* transcript levels in CP-Gal4>UAS-miR-275-TuD females. To verify this hypothesis, we injected 100 pmol of miR-275 mimic into CP-Gal4>UAS-miR-275-TuD and control female mosquitoes. Treatment with miR-275 mimic elevated the *SERCA* transcript level in guts of both wt and hybrid mosquitoes (Fig 4B). These results have shown that miR-275 targets and positively regulates *SERCA*.

### The effect of miR-275 on gut enzyme biosynthesis

**Fig 5:**
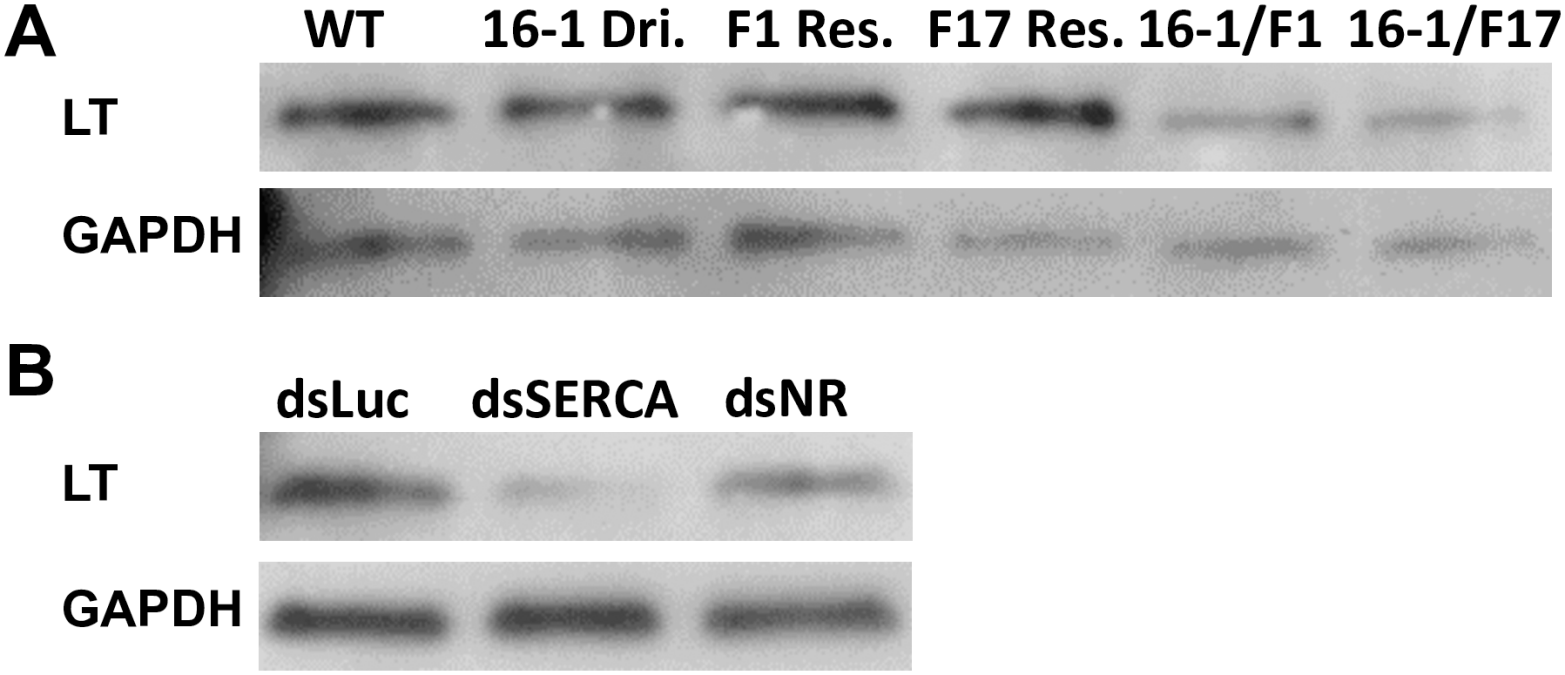
miR-275 affects late trypsin (LT) protein level in guts of female mosquitoes. (A) Western blot analyses using antibodies against LT in guts from CP-Gal4>UAS–miR-275-TuD (16-1/F1, 16-1/F17) female mosquitoes compared with wt, 16–1 Dri. (16–1 Driver), F1 Res. (F1 Responder) and F17 Res. (F17 Responder) female mosquitoes. GAPDH was used as a loading control. (B) SERCA RNAi (dsSERCA) and Notch receptor RNAi (dsNR) treated mosquitoes exhibited reduced LT protein levels.

Endoplasmic reticulum (ER) Ca^2+^ level is essential for protein synthesis, folding, transport and secretion, and its disruption results in disturbance of these functions [19]. Because miR-275 gut-specific depletion leads to a dramatic reduction of *SERCA* transcript level, we asked whether the miR-275 has a function related to secretion of digestive enzymes. We focused our attention on late trypsin (LT), which is an essential late-phase digestive protease in guts of female mosquitoes [20]. To test whether LT production is affected, LT protein levels were evaluated at 24 h PBM in the female gut by means of Western blot analysis. LT levels in CP-Gal4>UAS-miR-275-TuD hybrid female guts were lower than in controls (Fig 5A), revealing that miR-275 is needed for LT protein production.

We then examined whether the LT secretion defect is due to the low *SERCA* gut level in the hybrid females. RNA interference (RNAi)-mediated knockdown of *SERCA* (dsSERCA) mimicked this adverse LT phenotype in guts of wt mosquitoes when compared with double-stranded RNA of *Luciferase* (dsLuc)-treated female mosquitoes (Fig 5B). RNAi depletion of Notch receptor (NR) caused a weaker reduction of the LT protein level (Fig 5B).

### TuD depletion of miR-275 affects Notch signaling and actin assembly in the mosquito gut

**Fig 6:**
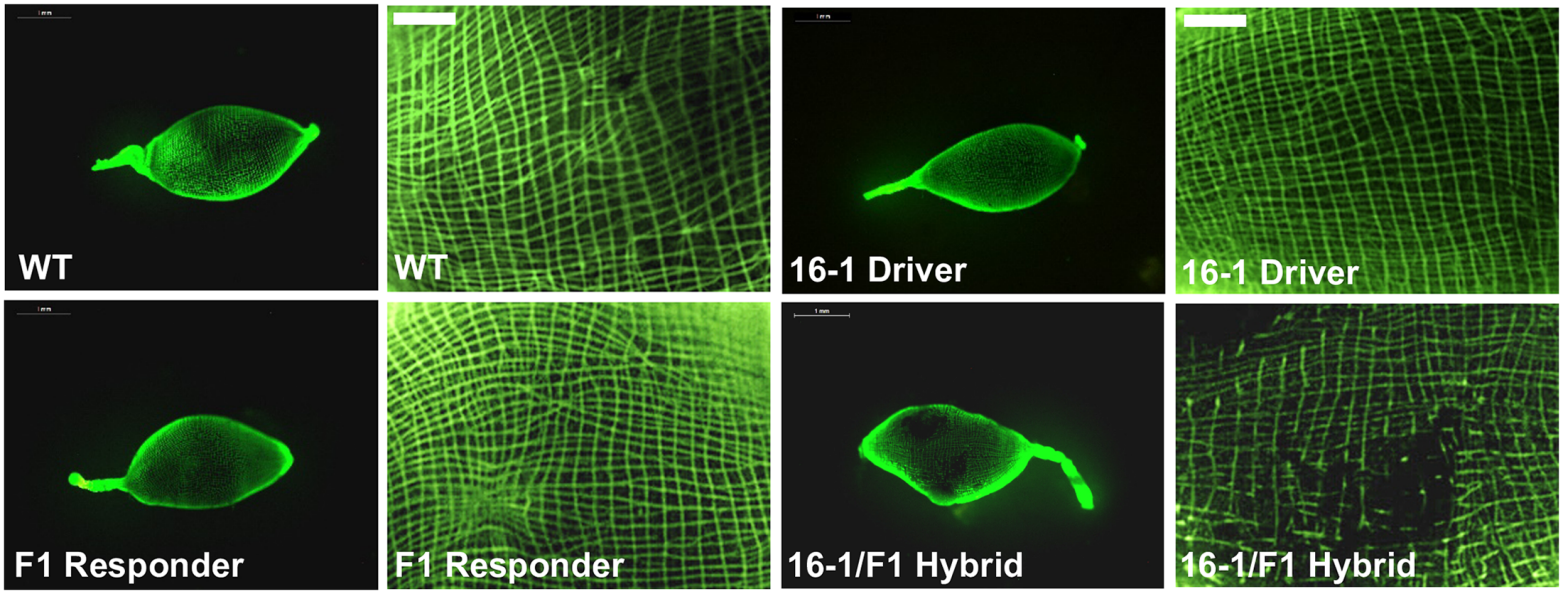
Depletion of miR-275 in CP-Gal4>UAS-miR-275-TuD female mosquitoes resulted in defects of the actin cytoskeleton. Actin cytoskeleton was destroyed in CP-Gal4>UAS-miR-275-TuD (16-1/F1) females compared with wt, 16–1 driver and F1 responder females.

**Fig 7:**
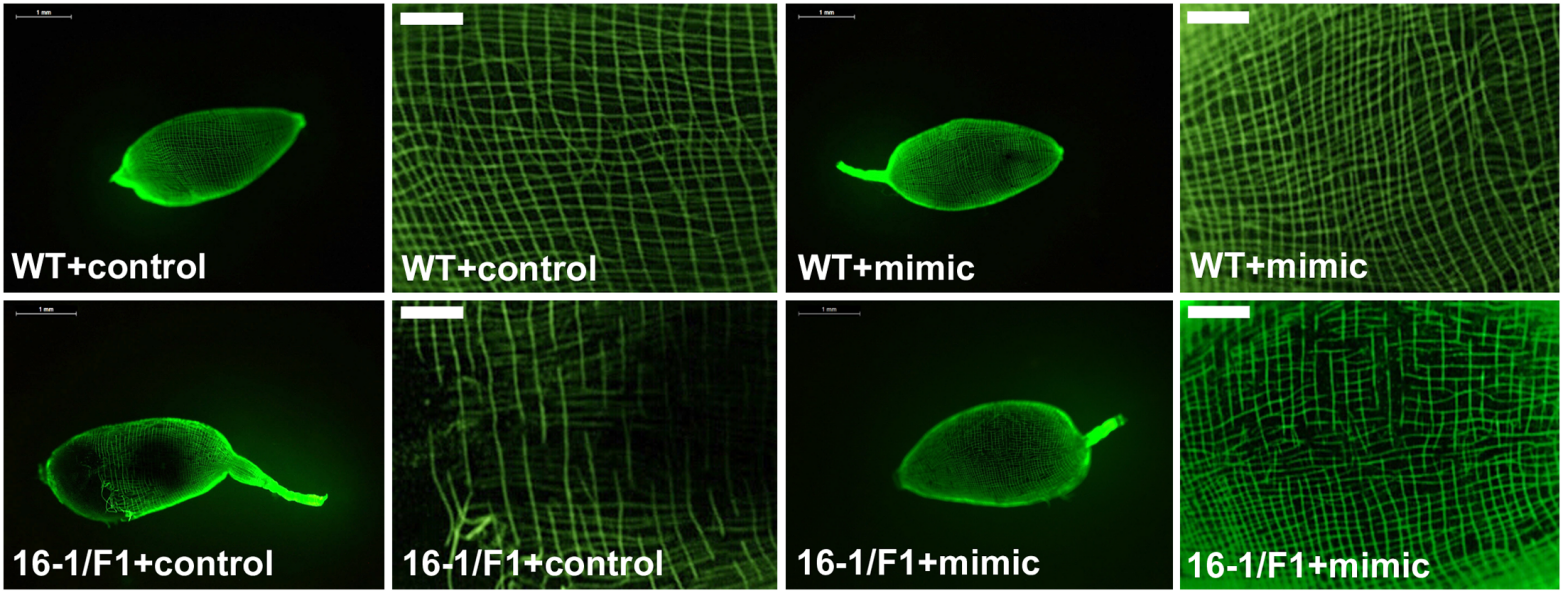
Actin cytoskeleton was partially rescued after miR-275 mimic application. Whole gut images show that the structure of the gut actin cytoskeleton was disturbed in CP-Gal4>UAS–miR-275-TuD (16-1/F1) female mosquitoes; miR-275 mimic rescued the actin cytoskeleton phenotype (Scale bar for gut actin staining: 0.2 mm (gut section); scale bar for whole gut actin staining: 1 mm (whole gut).

SERCA is a major determinant of the ER luminal protein synthesis and transport environment, which is critical for Notch signaling [21, 22]. *Drosophila* SERCA mutants failed to transport Notch receptor to the cell surface [22]. In humans, the SERCA inhibitor disrupts Notch signaling [13]. Based on pharmacological and genetic studies, the Notch signaling has been proposed to be vital for controlling actin cytoskeleton assembly [23–25]. In order to determine the effect of miR-275 and SERCA depletions on actin assembly, we visualized actin cytoskeleton in the mosquito gut by means of Actin staining. A well-organized cytoskeleton was observed in guts of wt female mosquitoes as well as in the driver and responder lines (Fig 6, S8 Fig). However, the actin cytoskeleton was disrupted in CP-Gal4>UAS-miR-275-TuD hybrid female guts, as has previously been observed with the application of systemic antagomir depletion of miR-275 [12] (Fig 6, S8 Fig). Injecting CP-Gal4>UAS-miR-275-TuD hybrid females with miR-275 mimic partially rescued this phenotype, indicating that the actin cytoskeleton defect was due to the miR-275 depletion (Fig 7, S8 Fig).

**Fig 8:**
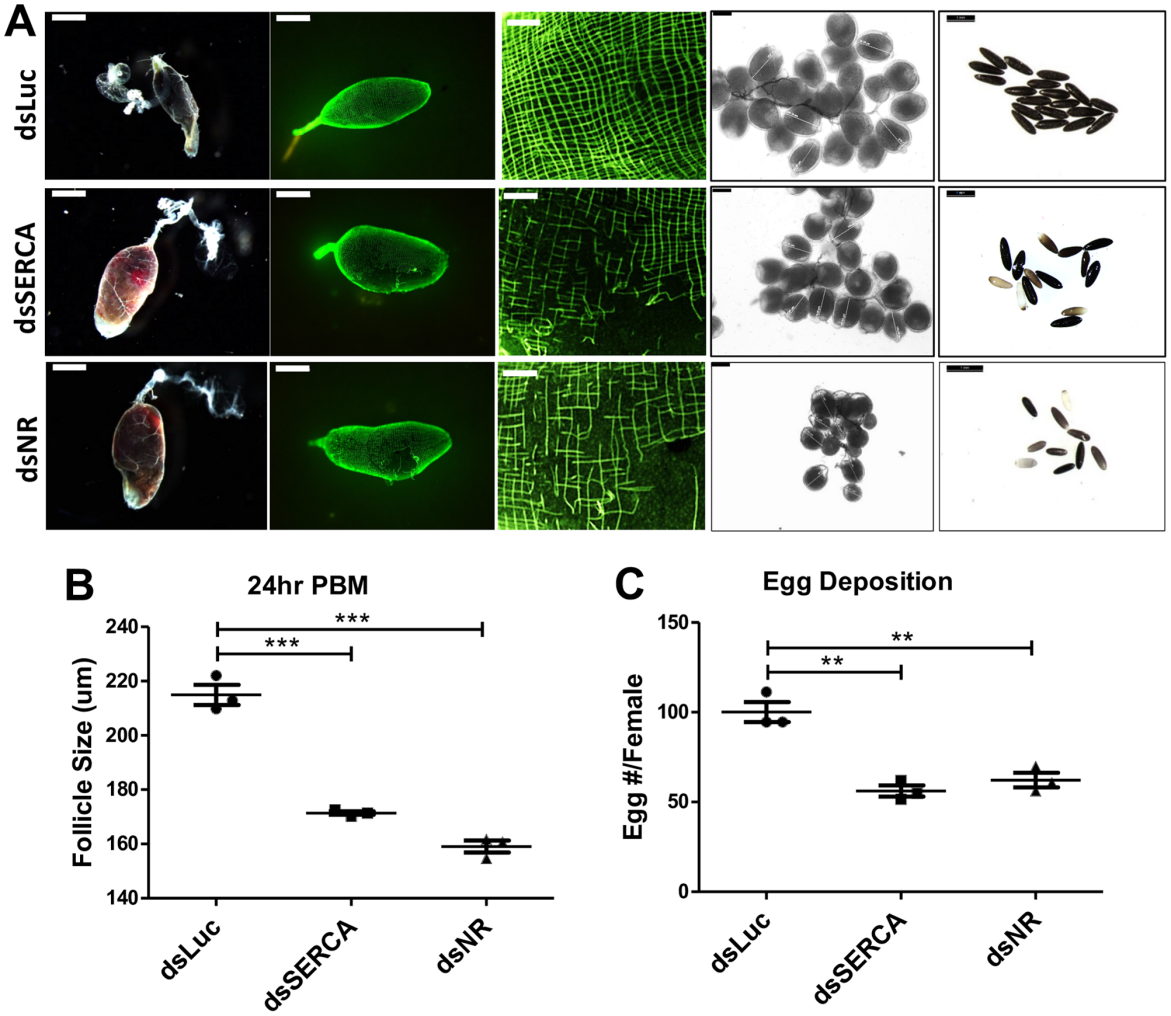
SERCA and NR RNAi mimics miR-275–depletion phenotypes. Female mosquitoes were injected with dsSERCA or dsNR, or dsLuc. SERCA and NR dsRNA-treated mosquitoes represent highly similar phenotypes to CP-Gal4>UAS–miR-275-TuD female mosquitoes. Images of guts, actin staining and ovaries were obtained at 24h PBM from under the Leica M165FC stereomicroscope (scale bar for whole gut actin staining and eggs: 1 mm; Scale bar for partial gut actin staining: 0.2 mm; scale bar for ovaries: 100 μm). (B) Average follicle size at 24h PBM after knockdown of SERCA or NR. (C) Knockdown of SERCA or NR reduced the average egg number. Values represent average ±s.e.m. from three combined biological replicates; **P < 0.01; ***P < 0.001.

We examined whether this defect is due to the low SERCA gut level in the hybrid females caused by miR-275 silencing. Abnormal actin cytoskeleton was observed in guts of wt mosquitoes after SERCA RNAi-mediated knockdown (Fig 8). Moreover, SERCA RNAi resulted in other phenotypes characteristic of miR-275 depletion (Fig 8). To validate the observed phenotypes in *SERCA* knocked-down mosquitoes, we chose thapsigargin (TG), a highly specific inhibitor of SERCA [26]. Injections of TG inhibited blood digestion, actin cytoskeleton assembly, follicle development, and egg-deposition production phenotypes observed in CP-Gal4>UAS–miR-275-TuD females and dsSERCA-treated mosquitoes (S9A–S9C Fig). To establish involvement of Notch signaling, we utilized *Notch receptor* RNAi, which showed the same phenotype of actin disruption as SERCA RNAi silencing (Fig 8). Moreover, *Notch receptor* RNAi also led to the same set of adverse responses, including an apparent lack of blood digestion, inhibition of egg development and reduced egg number compared with controls (Fig 8). For further *in vivo* analysis, we used DAPT, a γ-secretase inhibitor of Notch signaling [26, 27], to validate the observed phenotypes in *Notch receptor* (*NR*) knocked-down mosquitoes. Injection of DAPT exhibited phenotypes highly similar to CP-Gal4>UAS-miR-275-TuD mosquitoes (S9A–S9C Fig). Hence, our data suggest that the Notch receptor is a downstream effector of miR-275 and SERCA in the assembly of actin cytoskeleton in the mosquito gut.

### miR-275 plays a critical role in the maintenance of gut microbiota

**Fig 9:**
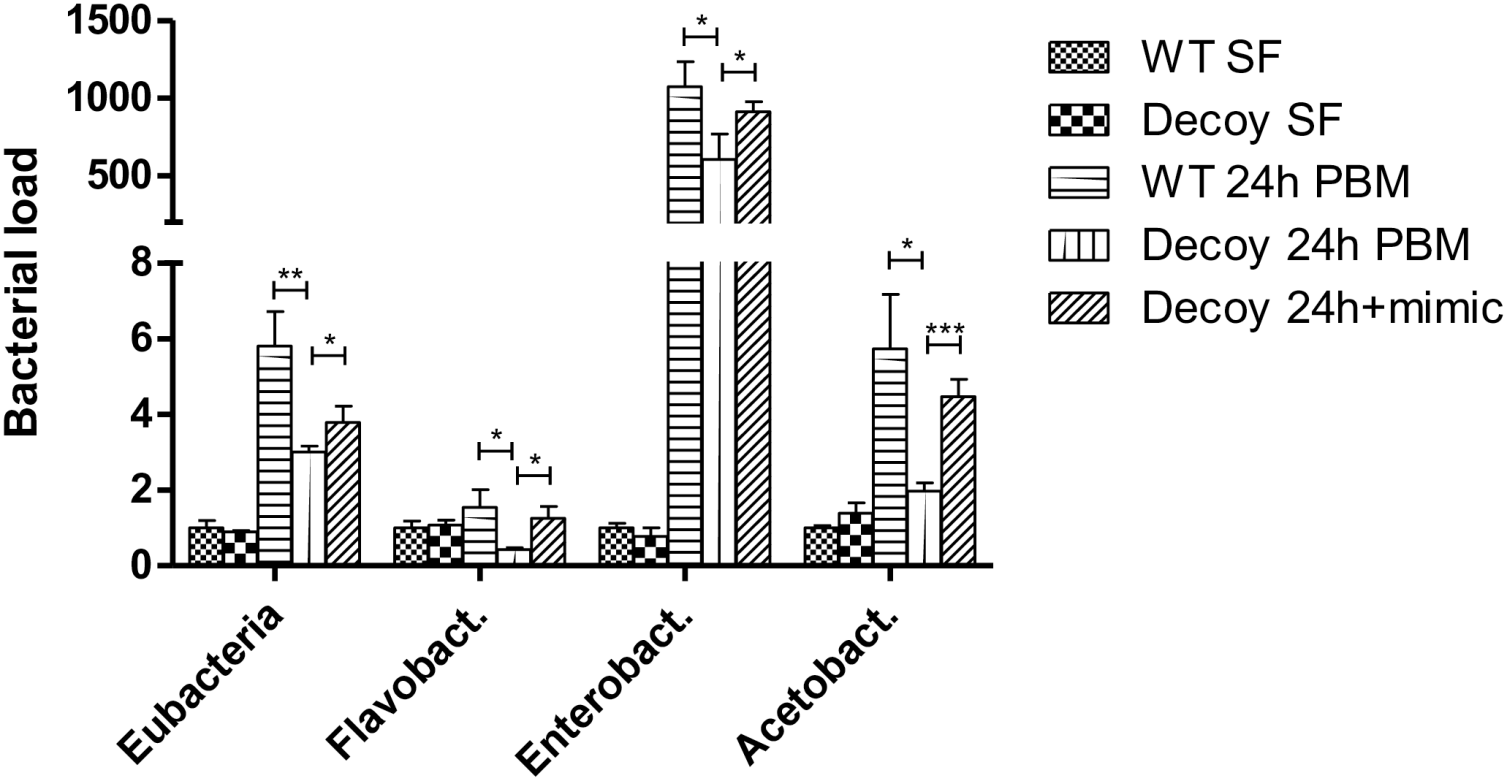
Effect of miR-275 silencing on mosquito gut microbiota. q-PCR quantification of gut bacterial 16S rDNA at sugar-fed stage (SF) and at 24 h post blood meal (PBM). q-PCR microbiota analysis using generic or taxon-specific 16S rDNA primers. Bacterial population was less in CP-Gal4>UAS–miR-275-TuD female mosquitoes (Decoy) than in wt. miR-275 mimic application partially rescued the level of gut microbiota. Enterobact.—Enterobacteriaceae; Flavobact.—Flavobacteriaceae; Acetobact.—Acetobacteraceae. Values represent average ±s.e.m. from three combined biological replicates; 15–25 mosquitoes per sample; *P < 0.05; **P < 0.01; ***P < 0.001.

In mosquitoes, commensal gut microbiota proliferates in response to a blood meal [28]. It is required for blood digestion, nutrition and immune defense [29]. Because of the profound effect of miR-275 depletion on the mosquito gut, we examined the level of four families of gut microbiota (*Eubacteria*, *Enterobacteriaceae*, *Flavobacteriaceae* and *Acetobacteraceae*) using q-PCR. We quantified bacterial content in the gut at the sugar-fed stage (SF) and 24 h PBM in wt and CP-Gal4>UAS–miR-275-TuD females. q-PCR analysis using generic or taxon-specific 16S rDNA primers revealed that the massive proliferation of bacteria seen at 24 h PBM was significantly less in miR-275-depleted mosquitoes than in wt controls (Fig 9). This inhibitory effect was partly removed by miR-275 mimic injection in CP-Gal4>UAS–miR-275-TuD females (Fig 9). *Acetobacteraceae* were particularly sensitive to the diminished level of miR-275 in the gut.

## Discussion

Sequence-specific antisense TuD inhibitors represent an effective means for targeted miRNA suppression [30–32]. In this work, we utilized the TuD approach combined with the Gal4/UAS system for spatiotemporal silencing of miR-275 in the mosquito gut. Compared with levels in control lines, miR-275 levels in guts of both CP-Gal4>UAS-miR-275-TuD mosquito lines significantly decreased after blood feeding, the time when the *CP* promoter was activated and caused overexpression of miR-275-TuD RNA. Depletion of miR-275 by gut-specific expression of the miR-275 TuD RNA resulted in dramatic defects in blood digestion, gut structure and, as a consequence, egg development. These defects are consistent with results reported previously using systemic inactivation of miR-275 by antagomir injection [12]. However, utilization of the CP-Gal4>UAS-miR-275-TuD system eliminated the possibility of miR-275 depletion effects in other tissues. Our tests have clearly shown that the observed phenotypes are due to miR-275 suppression in the gut.

Analysis using five computational programs identified the mosquito *SERCA* orthologue as a potential target of miR-275. The luciferase reporter assay validated the *SERCA* 3'-UTR response to miR-275 *in vitro* as its authentic target. It is supported by miR-275 mimic application, which partially restored the *SERCA* transcript level in guts of miR-275-TuD mosquitoes. Because depletion of miR-275 results in a decrease of *SERCA* transcript level, it appears that miR-275 controls either *SERCA* transcription or stability of its mRNA. It has been established that miRNAs inhibit protein synthesis by either negatively regulating the expression and subsequent degradation of mRNA targets or repressing translation [4, 33]. However, recent studies have also suggested that some miRNAs can up-regulate particular target mRNA or activate mRNA translation [34–38]. One possibility is that such amiRNA may have the ability to stabilize mRNA or activate translation by recruiting specific proteins that could lead to the accumulation of the mRNA. For instance, miR369-3 forms the AGO2-FXR1 complex at the mRNA binding site [34]. It has been reported that, in *A*. *aegypti* mosquitoes, aae-miR-375 expression upregulates the transcript level of the Toll immune pathway component *Cactus* [7]. Another study demonstrated that *Wolbachia* uses a host microRNA, aae-miR-2940, to enhance transcript levels and/or the stability of the mRNA of *m41 ftsh* in *A*. *aegypti* [6].

SERCA is a conserved transmembrane ion transporter of the P(II)-type ATPase family that actively transports Ca^2+^ from the cytosol to the sarco/endoplasmic reticulum stores against a large gradient concentration [21, 39]. It is vital for normal functioning and integrity of the muscle tissue [21, 40, 41]. In agreement with this, we observed disruptions in the actin cytoskeleton of gut walls of CP-Gal4>UAS-miR-275-TuD mosquitoes. Sustaining optimal Ca^2+^ concentration in the ER lumen by SERCA is critical for protein synthesis and transport [22]. We detected diminished LT levels in guts of CP-Gal4>UAS-miR-275-TuD mosquitoes, suggesting a decrease in the overall digestive enzyme production as a result of miR-275 and *SERCA* silencing. This in turn leads to interruption of normal blood digestion and, as a consequence, retardation of egg development.

In addition, we observed disruption of the gut microbiota proliferation in CP-Gal4>UAS-miR275-TuD mosquitoes following the blood meal, demonstrating the importance of miR-275 and SERCA in its maintenance. This link is further supported by rescue experiments with miR-275 mimic. Microbiota misbalance could occur as a result of Ca^2+^ disturbance in CP-Gal4>UAS-miR-275-TuD mosquitoes. However, other gut functions disrupted by miR-275 depletion, such as digestive enzyme secretion, could also contribute to the health of gut symbiotic bacteria, although a recent report on *Drosophila* indicated that alteration in their microbiota impairs gut function [42]. Thus, it is possible that miR-275-dependent gut microbiota homeostasis is essential for maintaining normal gut performance. Further studies should clarify this.

One pronounced phenotype observed in mosquitoes with systemic [12] or transgenic TuD silencing of miR-275 is disruption of gut actin cytoskeleton assembly. In CP-Gal4>UAS-miR-275-TuD mosquitoes, the actin cytoskeleton integrity was rescued by the miR-275 mimic application, indicating miR-275 involvement in the actin cytoskeleton gut assembly. Treatment of wt mosquitoes with either dsSERCA or the SERCA inhibitor TG created the same phenotype as miR-275 silencing, confirming that this miRNA targets SERCA and, in turn, SERCA is required for the actin cytoskeleton integrity. It has been reported that maintaining high Ca^2+^ concentration in the ER is essential for Notch signaling protein transport in *Drosophila* and humans [13, 22]. In the SERCA mutants, Notch cleavage and receptor trafficking to the cell membrane are impaired [22]. TG inhibition of SERCA interferes with early maturation of Notch1 in the ER [13]. Moreover, Notch signaling has been implicated as a regulator in actin assembly [23]. Recently, miR-34/449 was shown to control actin network formation by repressing cell cycle genes and the Notch signaling pathway [25]. Our data have also implicated the Notch signaling in maintaining the integrity of the gut actin cytoskeleton. The disruption of Notch signaling by either *Notch receptor* RNAi silencing or application of the Notch signaling inhibitor DAPT adversely affects the gut actin cytoskeleton in a manner similar to the action of miR-275-TuD or dsSERCA RNAi silencing. This suggests that miR-275 affects the gut actin cytoskeleton by its control of SERCA, the normal functioning of which is required for Notch signaling and, in turn, actin cytoskeleton assembly.

Recent studies have implicated miRNAs as regulators of Ca^2+^ homeostasis. miR-22 has been shown to control Ca^2+^ homeostasis in mammalian cardiac muscle tissue [14]. Genetic ablation of miR-22 in mice led to a decrease of the *SERCA* transcript levels and reduced ER load of Ca^2+^. miR-22 appears to act on SERCA indirectly affecting its target, the transcriptional/translational repressor purine-rich element binding protein B [14]. In this study, we have discovered that miR-275 directly targets SERCA, controlling multiple key functions in the mosquito gut. Further studies are required to identify miRNAs directly affecting SERCA in vertebrates and humans.

## Materials and methods

### Animals

*A*. *aegypti* UGAL strain (wt) mosquitoes were used in this study. The wt and transgenic strains were maintained at 27°C, with 80% relative humidity. Adults were reared with supply of 10% (wt/vol) sucrose solution and water. Three or four days after eclosion, the female mosquitoes were fed on the blood of White Leghorn chickens. The use of vertebrate animals was approved by the University of California Riverside IACUC.

### Plasmid construction

The AAEL-UAST_Decoy-linker-aae-mir-275-3P (t5) (UAS-miR-275-TuD construct) was a kind gift from Dr. Chun-Hong Chen. The UAS-miR-275-TuD construct was used as a responder plasmid in transgenesis and was produced based on the UAS-EGFP Responder [43], with the EGFP coding region being replaced by the miR-275-TuD cassette (S2 Fig).

### Germ-line transformation of *A*. *aegypti*

The responder plasmid (UAS-miR-275-TuD construct) and phsp-pBac helper plasmids were purified using the EndoFree Plasmid Maxi Kit (QIAGEN). Responder (0.35 mg/ml) and helper (0.25 mg/ml) plasmids were re-suspended in injection buffer (pH 6.8, containing 5mM KCl), and the mixture was then injected into pre-blastoderm stage eggs. The development of transgenic lines was performed as described previously [43]. G1 progeny was screened for the presence of dsRed fluorescent eye marker. The CP-Gal4>UAS-miR-275-TuD hybrid lines were established as described previously [43]. Hybrid mosquitoes exhibited EGFP and dsRed eye-specific selectable markers. In all screening experiments, a Nikon SMZ800 fluorescence microscope fitted with dsRed and GFP-B filter sets was used.

### Molecular analysis

DNeasy tissue kit (QIAGEN) was used to extract genomic DNA from adult mosquitoes. A total of 200 ng genomic DNA was used as a template in PCR on the F1-1 and F17-1 responder lines using the Platinum PCR SuperMix (Invitrogen). The PCR was performed by using specific primers (S2 Table), at 94°C for 2 min, followed by 35 cycles of 94°C for 30 s, 55°C for 30 s, 68°C for 30 s, and a final extension at 68°C for 7 min. The miR-275 level was examined quantitatively using qRT-PCR. QIAGEN miScript reverse transcription kits and miScript SYBR Green PCR kits were used to perform qRT-PCR according to manufacturer’s protocols. Using TRIzol (Invitrogen), total RNA was isolated from 10 guts of blood-fed female mosquitoes at different time points. A 1-μg sample of total RNA was treated by DNase I and subjected to cDNA production using the miScript reverse transcription kit. cDNA was checked using the miScript SYBR Green PCR kit (qRT-PCR) under the following conditions: 95°C for 15 min, and 40 cycles of 94°C for 15 s, 60°C for 30 s and 70°C for 30 s, followed by the melting curve (65°C-95°C). The *ribosomal protein S7* (*RPS7*) was chosen as an internal control gene. Analysis is as relative level to *RPS7*, as described previously [12]. Injection of miR-275 mimic, control mimic and double-stranded RNA (dsRNA) was carried out in 1-day-old cold anesthetized female mosquitoes. A specific region of the *SERCA* gene was amplified by PCR using gene-specific primers (S2 Table). The PCR product was used as a template to synthesize dsRNA using the MEGAscript kit (Ambion). Mosquitoes were injected with 0.5 μL dsRNA at a concentration of 4 μg/μL and 0.5 μL mimic at a concentration of 200 μM. dsRNA from a region of *Luciferase* gene (dsLuc) was used as negative control. Mosquitoes were blood fed 3–4 days after injection.

### miRNA target studies

Computational target prediction was performed as previously described [44]. Five programs—miRANDA, PITA, TargetScan, RNAhybrid and a program developed in-house—were used to find potential targets of miR-275 in the *A*. *aegypti* genome [44]. *In vitro* target validation was performed as previously described [44]. miR-275 mimic (5’-UCAGGUACCUGAAGUAGCGC-3’) and control mimic (5’-UCACAACCUCCUAGAAAGAGUAGA-3’) were synthesized by QIAGEN. *Drosophila* Schneider S2 (S2) cells (Invitrogen) were maintained at 28°C in Schneider’s *Drosophila* medium (Gibco) with 1× Antibiotic-Antimycotic (Gibco) containing 10% heat-inactivated fetal bovine serum (Gibco). We used a Dual Luciferase Reporter Assay System (Promega). The transfection was performed in S2 cells using the Attractene reagent (QIAGEN). In each transfection, 100 ng of psiCheck-2 reporters and synthetic miR-275 mimic or control mimic at a final concentration of 100 nm were used. A no-mimic treatment was performed at the same time in each transfection. The expression of luciferase was quantified by measuring luminescence activities 48 h post-transfection. Firefly luciferase was used for normalization of *Renilla* luciferase expression. Three biological replicates, each with three technical replicates, were performed. The SERCA inhibitor TG was purchased from Alomone labs and prepared as a 100-mM stock solution in DMSO—0.5 μL of the working solution at a concentration of 250 μM was injected into mosquitoes after blood meal. The Notch signaling inhibitor DAPT was purchased from Sigma and prepared as a 10-mM stock solution in DMSO, and 50 μM was used as the working concentration.

### Actin staining

24 hours PBM, mosquitoes were cold anesthetized and intra-thoracically injected with staining solution and allowed to incubate for 30 min. The staining solution contains 0.165 μM phalloidin Alexa Fluor 488 (A12379; Invitrogen), 1% Triton-X 100, and 8% formaldehyde in APS (Aedes physiological saline) [12]. Guts were dissected and washed in APS-T (APS with 0.3% Triton-X 100) and mounted using VECTASHIELD Mounting Medium (Vector Labs). All photographs were captured using a Leica M165FC fluorescent stereomicroscope and LAS V4.0 software. Images are representative of ten mosquitoes for each treatment.

### Western blot

Detection of LT and GAPDH was performed as previously described by Isoe J et al [20]. Guts from blood-fed female mosquitoes were homogenized in tissue extraction buffer (Invitrogen). Then, 10 μg protein was boiled in LDS (4×) NuPage sample buffer (Invitrogen) with 10× sample reducing agent (Invitrogen). For detection of LT, LT antibodies were used at a 1:200 dilution followed by the secondary anti-rabbit-HRP (Abcam) at a 1:5000 dilution. For detection of GAPDH, GAPDH monoclonal antibodies (Cell Signaling) were used at a 1:200 dilution followed by the secondary anti-mouse-HRP (Abcam) at a 1:5000 dilution.

### Microbiota analysis

Microbiota analysis using q-PCR was performed according to Gendrin et al [29]. Every sample contained either 15 non-blood-fed or blood-fed mosquito guts. Samples were homogenized with disposable pestles (USA Scientific). DNA was extracted using a tissue and blood DNA extraction kit (Qiagen). The 16S rDNA is shown as a ratio of the *RPS7*. q-PCR was performed on a CFX-96 Real-Time thermocycler (Bio-Rad) using the SYBR mix (Bio-Rad), following the manufacturer’s instructions.

## Supporting information

S1 Fig Details of the constructs for transgenesis. (A) Schematic representation of the driver CP-Gal4 cassette. (B) Schematic representation of the responder UAS-miR-275-TuD cassette. (C) Schematic representation of the miR-275-TuD RNA.
(TIF)

S2 Fig Partial sequence of the UAS-miR-275-TuD construct. (TIF)

S3 Fig Confirmation of the CP-Gal4>UAS-miR-275-TuD hybrid transgenic mosquito lines. (A-D) Transgenic CP-Gal4/UAS–miR-275-TuD hybrids present both selectable markers. Pupae of mosquitoes were screened for the presence of EGFP and dsRed-selectable markers in the eye. Pupae were visualized under the Leica M165FC stereomicroscope, and images were obtained using a GFP-B filter or dsRed filter and LAS V4.0 software. Scale bar: 1 mm. (E) Genomic PCR was used to confirm stable incorporation and the integrity of UAS-miR-275-TuD construct in the CP-Gal4>UAS-miR-275-TuD hybrid transgenic mosquito lines (16-1/F1, 16-1/F17). (F) miR-275 depletion is tissue-specific. miR-275 levels were not affected in the fat body at 24 h PBM in CP-Gal4>UAS–miR-275-TuD (16-1/F1, 16-1/F17) female mosquitoes. Values represent average ±s.e.m. from three combined biological replicates.
(TIF)

S4 Fig Effect of gut-specific depletion of miR-275 in blood-fed *A*. *aegypti* female mosquitoes. (A) Female mosquito ovaries at 24 h PBM, and eggs (B) produced by multiple mosquito lines. These lines include wt, 16–1 Driver, F1 Responder, and CP-Gal4>UAS–miR-275-TuD lines (16-1/F1). Images of ovaries and eggs were captured from under the Leica M165FC stereomicroscope (scale bar for eggs: 1 mm; scale bar for ovaries: 100 μm).
(TIF)

S5 Fig miR-275 mimic rescues the phenotypes in blood-fed CP-Gal4>UAS–miR-275-TuD female mosquitoes. Female mosquito ovaries were sampled at 24 h PBM; eggs produced by multiple mosquito lines (Scale bar for eggs: 1 mm; scale bar for ovaries: 100 μm). (A) miR-275 mimic rescued the phenotype of reduced follicle size. (B) miR-275 mimic rescued the phenotype of aberrant egg shape.
(TIF)

S6 Fig miR-275 mimic rescues the phenotypes in blood-fed CP-Gal4>UAS–miR-275-TuD female mosquitoes (16-1/F17). Female mosquito guts and ovaries were sampled at 24 h PBM (Scale bar for guts and eggs: 1 mm; scale bar for ovaries: 100 μm).
(TIF)

S7 Fig Schematic representation of the construct psiCHECK-2-SERCA and psiCHECK-2-ΔSERCA. (A) psiCHECK-2-SERCA contains 3’UTR from SERCA which includes a predicted miR-275 binding site. (B) In the psiCHECK-2-ΔSERCA construct, the predicted miR-275 binding site was mutated.
(TIF)

S8 Fig Actin cytoskeleton was partially rescued in CP-Gal4>UAS-miR-275-TuD (16-1/F17) female mosquitoes after miR-275 mimic application. (Scale bar for gut actin staining: 0.2 mm (partial gut); scale bar for whole gut actin staining: 1 mm (whole gut).
(TIF)

S9 Fig Application of SERCA-specific inhibitor TG and Notch signaling specific inhibitor DAPT resulted in phenotypes similar to CP-Gal4>UAS-miR-275-TuD (16-1/F1, 16-1/F17) female mosquitoes. (A) Phenotypes of mosquito guts, ovaries and eggs after TG or DAPT treatment. Photos of guts and ovaries were captured at 24 h PBM. Actin staining was performed at 24 h PBM. (Scale bar for eggs: 1 mm; Scale bar for whole gut actin staining: 1 mm; Scale bar for partial gut: 0.2 mm; Scale bar for ovaries: 100 μm). (B) Average follicle sizes were dramatically reduced at 24h PBM in mosquitoes after TG or DAPT treatment. (C) Injection of TG or DAPT reduced the average egg number. Images of guts, actin staining, ovaries and eggs were captured using the Leica M165FC stereomicroscope. Values represent average ±s.e.m. from three combined biological replicates; *P < 0.05; **P < 0.01; ***P < 0.001.
(TIF)

S1 Table miR-275 target prediction in *Ae*. *aegypti* 3′ UTRs. (DOCX)

S2 Table Primer sequences. (DOCX)

## References

[1] Akbari OS, Antoshechkin I, Amrhein H, Williams B, Diloreto R, Sandler J, et al The developmental transcriptome of the mosquito Aedes aegypti, an invasive species and major arbovirus vector. G3. 2013;3(9):1493–509. doi: 10.1534/g3.113.006742 . 23833213 10.1534/g3.113.006742 PMC3755910

[2] Roy S, Saha TT, Johnson L, Zhao B, Ha J, White KP, et al Regulation of Gene Expression Patterns in Mosquito Reproduction. PLoS genetics. 2015;11(8):e1005450 doi: 10.1371/journal.pgen.1005450 . 26274815 10.1371/journal.pgen.1005450 PMC4537244

[3] Lucas KJ, Zhao B, Liu S, Raikhel AS. Regulation of physiological processes by microRNAs in insects. Current opinion in insect science. 2015;11:1–7. doi: 10.1016/j.cois.2015.06.004 . 26251827 10.1016/j.cois.2015.06.004 PMC4522942

[4] Djuranovic S, Nahvi A, Green R. miRNA-mediated gene silencing by translational repression followed by mRNA deadenylation and decay. Science. 2012;336(6078):237–40. doi: 10.1126/science.1215691 . 22499947 10.1126/science.1215691 PMC3971879

[5] Fabian MR, Sonenberg N, Filipowicz W. Regulation of mRNA translation and stability by microRNAs. Annual review of biochemistry. 2010;79:351–79. doi: 10.1146/annurev-biochem-060308-103103 . 20533884 10.1146/annurev-biochem-060308-103103

[6] Hussain M, Frentiu FD, Moreira LA, O'Neill SL, Asgari S. Wolbachia uses host microRNAs to manipulate host gene expression and facilitate colonization of the dengue vector Aedes aegypti. Proceedings of the National Academy of Sciences of the United States of America. 2011;108(22):9250–5. doi: 10.1073/pnas.1105469108 . 21576469 10.1073/pnas.1105469108 PMC3107320

[7] Hussain M, Walker T, O'Neill SL, Asgari S. Blood meal induced microRNA regulates development and immune associated genes in the Dengue mosquito vector, Aedes aegypti. Insect biochemistry and molecular biology. 2013;43(2):146–52. doi: 10.1016/j.ibmb.2012.11.005 . 23202267 10.1016/j.ibmb.2012.11.005

[8] Lucas KJ, Myles KM, Raikhel AS. Small RNAs: a new frontier in mosquito biology. Trends in parasitology. 2013;29(6):295–303. doi: 10.1016/j.pt.2013.04.003 . 23680188 10.1016/j.pt.2013.04.003 PMC5739026

[9] Haraguchi T, Nakano H, Tagawa T, Ohki T, Ueno Y, Yoshida T, et al A potent 2'-O-methylated RNA-based microRNA inhibitor with unique secondary structures. Nucleic acids research. 2012;40(8):e58 doi: 10.1093/nar/gkr1317 . 22259037 10.1093/nar/gkr1317 PMC3333889

[10] Elliott DA, Brand AH. The GAL4 system: a versatile system for the expression of genes. Methods in molecular biology. 2008;420:79–95. doi: 10.1007/978-1-59745-583-1_5 . 18641942 10.1007/978-1-59745-583-1_5

[11] Zhao B, Hou Y, Wang J, Kokoza VA, Saha TT, Wang XL, et al Determination of juvenile hormone titers by means of LC-MS/MS/MS and a juvenile hormone-responsive Gal4/UAS system in Aedes aegypti mosquitoes. Insect biochemistry and molecular biology. 2016;77:69–77. doi: 10.1016/j.ibmb.2016.08.003 . 27530057 10.1016/j.ibmb.2016.08.003 PMC5028310

[12] Bryant B, Macdonald W, Raikhel AS. microRNA miR-275 is indispensable for blood digestion and egg development in the mosquito Aedes aegypti. Proceedings of the National Academy of Sciences of the United States of America. 2010;107(52):22391–8. doi: 10.1073/pnas.1016230107 . 21115818 10.1073/pnas.1016230107 PMC3012520

[13] Roti G, Carlton A, Ross KN, Markstein M, Pajcini K, Su AH, et al Complementary genomic screens identify SERCA as a therapeutic target in NOTCH1 mutated cancer. Cancer cell. 2013;23(3):390–405. doi: 10.1016/j.ccr.2013.01.015 . 23434461 10.1016/j.ccr.2013.01.015 PMC3709972

[14] Gurha P, Abreu-Goodger C, Wang T, Ramirez MO, Drumond AL, van Dongen S, et al Targeted deletion of microRNA-22 promotes stress-induced cardiac dilation and contractile dysfunction. Circulation. 2012;125(22):2751–61. doi: 10.1161/CIRCULATIONAHA.111.044354 . 22570371 10.1161/CIRCULATIONAHA.111.044354 PMC3503489

[15] Asahi M, Sugita Y, Kurzydlowski K, De Leon S, Tada M, Toyoshima C, et al Sarcolipin regulates sarco(endo)plasmic reticulum Ca2+-ATPase (SERCA) by binding to transmembrane helices alone or in association with phospholamban. Proceedings of the National Academy of Sciences of the United States of America. 2003;100(9):5040–5. doi: 10.1073/pnas.0330962100 . 12692302 10.1073/pnas.0330962100 PMC154294

[16] Li S, Mead EA, Liang S, Tu Z. Direct sequencing and expression analysis of a large number of miRNAs in Aedes aegypti and a multi-species survey of novel mosquito miRNAs. BMC genomics. 2009;10:581 doi: 10.1186/1471-2164-10-581 . 19961592 10.1186/1471-2164-10-581 PMC2797818

[17] Zhao B, Kokoza VA, Saha TT, Wang S, Roy S, Raikhel AS. Regulation of the gut-specific carboxypeptidase: a study using the binary Gal4/UAS system in the mosquito Aedes aegypti. Insect biochemistry and molecular biology. 2014;54:1–10. doi: 10.1016/j.ibmb.2014.08.001 . 25152428 10.1016/j.ibmb.2014.08.001 PMC4426967

[18] Zhang Y, Zhao B, Roy S, Saha TT, Kokoza VA, Li M, et al microRNA-309 targets the Homeobox gene SIX4 and controls ovarian development in the mosquito Aedes aegypti. Proceedings of the National Academy of Sciences of the United States of America. 2016;113(33):E4828–36. doi: 10.1073/pnas.1609792113 . 27489347 10.1073/pnas.1609792113 PMC4995966

[19] Berridge MJ. The endoplasmic reticulum: a multifunctional signaling organelle. Cell calcium. 2002;32(5–6):235–49. . 12543086 10.1016/s0143416002001823

[20] Isoe J, Rascon AA Jr., Kunz S, Miesfeld RL. Molecular genetic analysis of midgut serine proteases in Aedes aegypti mosquitoes. Insect biochemistry and molecular biology. 2009;39(12):903–12. doi: 10.1016/j.ibmb.2009.10.008 . 19883761 10.1016/j.ibmb.2009.10.008 PMC2818436

[21] Hovnanian A. SERCA pumps and human diseases. Sub-cellular biochemistry. 2007;45:337–63. . 18193643 10.1007/978-1-4020-6191-2_12

[22] Periz G, Fortini ME. Ca(2+)-ATPase function is required for intracellular trafficking of the Notch receptor in Drosophila. The EMBO journal. 1999;18(21):5983–93. doi: 10.1093/emboj/18.21.5983 . 10545110 10.1093/emboj/18.21.5983 PMC1171664

[23] Giniger E. A role for Abl in Notch signaling. Neuron. 1998;20(4):667–81. . 9581760 10.1016/s0896-6273(00)81007-7

[24] Yeh TH, Huang SY, Lan WY, Liaw GJ, Yu JY. Modulation of cell morphogenesis by tousled-like kinase in the Drosophila follicle cell. Developmental dynamics: an official publication of the American Association of Anatomists. 2015;244(7):852–65. doi: 10.1002/dvdy.24292 . 25981356 10.1002/dvdy.24292

[25] Chevalier B, Adamiok A, Mercey O, Revinski DR, Zaragosi LE, Pasini A, et al miR-34/449 control apical actin network formation during multiciliogenesis through small GTPase pathways. Nature communications. 2015;6:8386 doi: 10.1038/ncomms9386 . 26381333 10.1038/ncomms9386 PMC4595761

[26] Treiman M, Caspersen C, Christensen SB. A tool coming of age: thapsigargin as an inhibitor of sarco-endoplasmic reticulum Ca(2+)-ATPases. Trends in pharmacological sciences. 1998;19(4):131–5. . 9612087 10.1016/s0165-6147(98)01184-5

[27] Imbimbo BP. Therapeutic potential of gamma-secretase inhibitors and modulators. Current topics in medicinal chemistry. 2008;8(1):54–61. . 18220933 10.2174/156802608783334015

[28] Pumpuni CB, Demaio J, Kent M, Davis JR, Beier JC. Bacterial population dynamics in three anopheline species: the impact on Plasmodium sporogonic development. The American journal of tropical medicine and hygiene. 1996;54(2):214–8. . 8619451 10.4269/ajtmh.1996.54.214

[29] Gendrin M, Rodgers FH, Yerbanga RS, Ouedraogo JB, Basanez MG, Cohuet A, et al Antibiotics in ingested human blood affect the mosquito microbiota and capacity to transmit malaria. Nature communications. 2015;6:5921 doi: 10.1038/ncomms6921 . 25562286 10.1038/ncomms6921 PMC4338536

[30] Haraguchi T, Ozaki Y, Iba H. Vectors expressing efficient RNA decoys achieve the long-term suppression of specific microRNA activity in mammalian cells. Nucleic acids research. 2009;37(6):e43 doi: 10.1093/nar/gkp040 . 19223327 10.1093/nar/gkp040 PMC2665227

[31] Bak RO, Hollensen AK, Mikkelsen JG. Managing microRNAs with vector-encoded decoy-type inhibitors. Molecular therapy: the journal of the American Society of Gene Therapy. 2013;21(8):1478–85. doi: 10.1038/mt.2013.113 . 23752312 10.1038/mt.2013.113 PMC3734669

[32] Bak RO, Hollensen AK, Primo MN, Sorensen CD, Mikkelsen JG. Potent microRNA suppression by RNA Pol II-transcribed 'Tough Decoy' inhibitors. Rna. 2013;19(2):280–93. doi: 10.1261/rna.034850.112 . 23249752 10.1261/rna.034850.112 PMC3543086

[33] Lucas KJ, Zhao B, Roy S, Gervaise AL, Raikhel AS. Mosquito-specific microRNA-1890 targets the juvenile hormone-regulated serine protease JHA15 in the female mosquito gut. RNA biology. 2015;12(12):1383–90. doi: 10.1080/15476286.2015.1101525 . 26488481 10.1080/15476286.2015.1101525 PMC4829293

[34] Vasudevan S, Tong Y, Steitz JA. Switching from repression to activation: microRNAs can up-regulate translation. Science. 2007;318(5858):1931–4. doi: 10.1126/science.1149460 . 18048652 10.1126/science.1149460

[35] Vasudevan S, Tong Y, Steitz JA. Cell-cycle control of microRNA-mediated translation regulation. Cell cycle. 2008;7(11):1545–9. doi: 10.4161/cc.7.11.6018 . 18469529 10.4161/cc.7.11.6018 PMC2556257

[36] Orom UA, Nielsen FC, Lund AH. MicroRNA-10a binds the 5'UTR of ribosomal protein mRNAs and enhances their translation. Molecular cell. 2008;30(4):460–71. doi: 10.1016/j.molcel.2008.05.001 . 18498749 10.1016/j.molcel.2008.05.001

[37] Henke JI, Goergen D, Zheng J, Song Y, Schuttler CG, Fehr C, et al microRNA-122 stimulates translation of hepatitis C virus RNA. The EMBO journal. 2008;27(24):3300–10. doi: 10.1038/emboj.2008.244 . 19020517 10.1038/emboj.2008.244 PMC2586803

[38] Niepmann M. Activation of hepatitis C virus translation by a liver-specific microRNA. Cell cycle. 2009;8(10):1473–7. doi: 10.4161/cc.8.10.8349 . 19372740 10.4161/cc.8.10.8349

[39] Bublitz M, Musgaard M, Poulsen H, Thogersen L, Olesen C, Schiott B, et al Ion pathways in the sarcoplasmic reticulum Ca2+-ATPase. The Journal of biological chemistry. 2013;288(15):10759–65. doi: 10.1074/jbc.R112.436550 . 23400778 10.1074/jbc.R112.436550 PMC3624456

[40] Periasamy M, Kalyanasundaram A. SERCA pump isoforms: their role in calcium transport and disease. Muscle & nerve. 2007;35(4):430–42. doi: 10.1002/mus.20745 . 17286271 10.1002/mus.20745

[41] Stammers AN, Susser SE, Hamm NC, Hlynsky MW, Kimber DE, Kehler DS, et al The regulation of sarco(endo)plasmic reticulum calcium-ATPases (SERCA). Canadian journal of physiology and pharmacology. 2015;93(10):843–54. doi: 10.1139/cjpp-2014-0463 . 25730320 10.1139/cjpp-2014-0463

[42] Clark RI, Salazar A, Yamada R, Fitz-Gibbon S, Morselli M, Alcaraz J, et al Distinct Shifts in Microbiota Composition during Drosophila Aging Impair Intestinal Function and Drive Mortality. Cell reports. 2015;12(10):1656–67. doi: 10.1016/j.celrep.2015.08.004 . 26321641 10.1016/j.celrep.2015.08.004 PMC4565751

[43] Kokoza VA, Raikhel AS. Targeted gene expression in the transgenic Aedes aegypti using the binary Gal4-UAS system. Insect biochemistry and molecular biology. 2011;41(8):637–44. doi: 10.1016/j.ibmb.2011.04.004 . 21536128 10.1016/j.ibmb.2011.04.004 PMC3124619

[44] Liu S, Lucas KJ, Roy S, Ha J, Raikhel AS. Mosquito-specific microRNA-1174 targets serine hydroxymethyltransferase to control key functions in the gut. Proceedings of the National Academy of Sciences of the United States of America. 2014;111(40):14460–5. doi: 10.1073/pnas.1416278111 . 25246546 10.1073/pnas.1416278111 PMC4209991

