# Supplementary figures and images for: MicroRNA-275 targets sarco/endoplasmic reticulum Ca2+ adenosine triphosphatase (SERCA) to control key functions in the mosquito gut
Source: PLoS Genet. 2017 Aug 7;13(8):e1006943. doi: 10.1371/journal.pgen.1006943 (PMC5560755; doi:10.1371/journal.pgen.1006943)

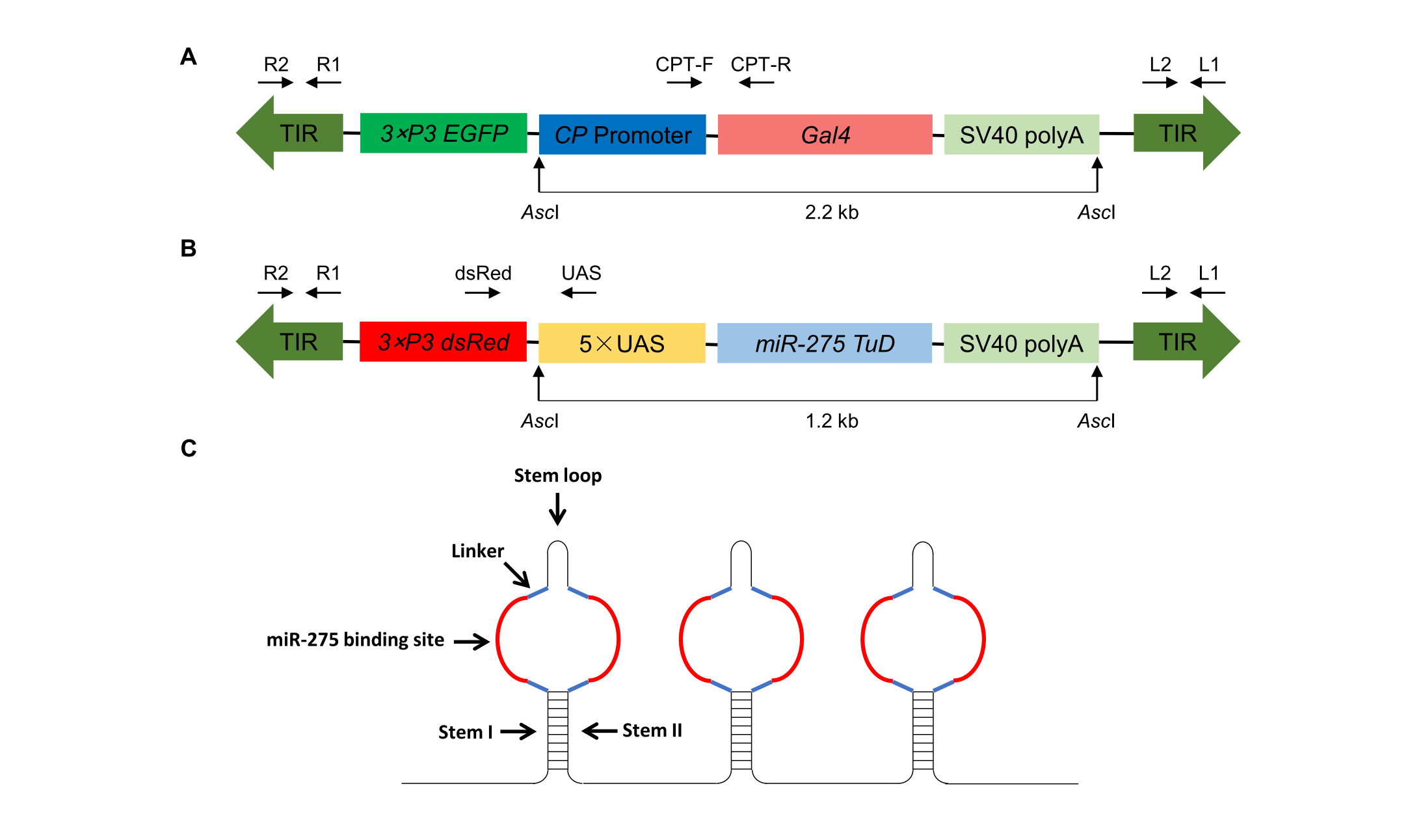

Supplement: S1 Fig — (A) Schematic representation of the driver CP-Gal4 cassette. (B) Schematic representation of the responder UAS-miR-275-TuD cassette. (C) Schematic representation of the miR-275-TuD RNA. (TIF) [file pgen.1006943.s001.tif]

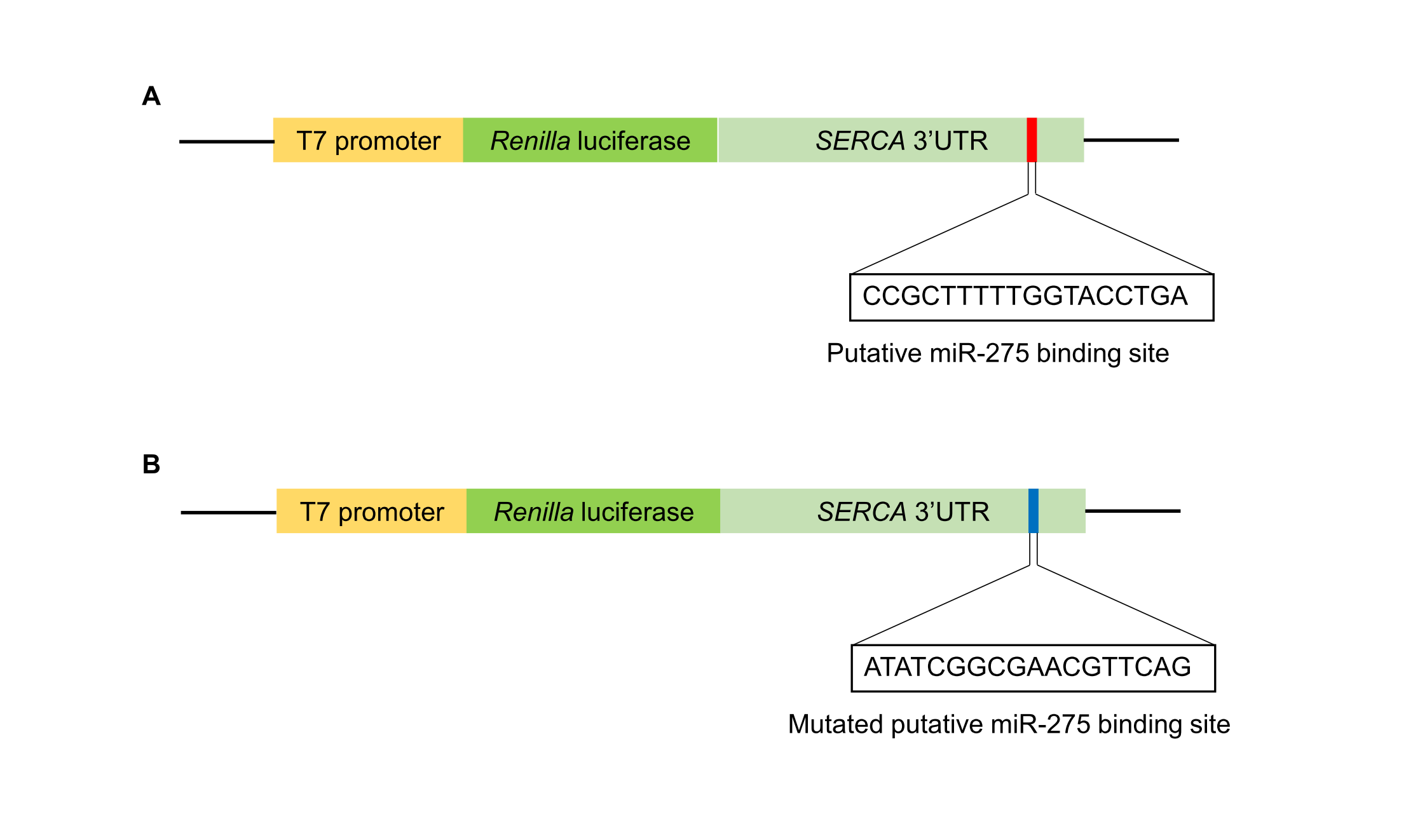

Supplement: S7 Fig — (A) psiCHECK-2-SERCA contains 3’UTR from SERCA which includes a predicted miR-275 binding site. (B) In the psiCHECK-2-ΔSERCA construct, the predicted miR-275 binding site was mutated. (TIF) [file pgen.1006943.s007.tif]
