# Supplementary material for: MicroRNA-275 targets sarco/endoplasmic reticulum Ca2+ adenosine triphosphatase (SERCA) to control key functions in the mosquito gut
Source: PLoS Genet. 2017 Aug 7;13(8):e1006943. doi: 10.1371/journal.pgen.1006943 (PMC5560755; doi:10.1371/journal.pgen.1006943)
Supplement: S1 Table — (DOCX) [file pgen.1006943.s010.docx]

Gene ID

Gene name

Program

AAEL006582

Sarco/endoplasmic reticulum Ca^2+^-ATPase

IN; TS; PITA; MR; RH

AAEL002714

Kinesin-like protein KIF23

IN; PITA; MR; RH

AAEL005191

cdk10/11 (cell division protein kinase 10/11)

IN; PITA; MR; RH

Programs used in target prediction: in-house (IN), TargetScan (TS), PITA; miRanda (MR), and RNAhybrid (RH).
