# Supplementary material for: MicroRNA-275 targets sarco/endoplasmic reticulum Ca2+ adenosine triphosphatase (SERCA) to control key functions in the mosquito gut
Source: PLoS Genet. 2017 Aug 7;13(8):e1006943. doi: 10.1371/journal.pgen.1006943 (PMC5560755; doi:10.1371/journal.pgen.1006943)
Supplement: S2 Table — (DOCX) [file pgen.1006943.s011.docx]

Gene (PCR) Sequence 5’-3’

*Actin*

Forward AAGGCCAACCGTGAGAAGATGACT

Reverse GCTCGTTGCCAATGGTGATGAC

*CP-Gal4*

Forward AGCGGAGACCTTTTGGTTTTG

Reverse TCCGACTTCACCCTACTGTTCTTT

*DsRed-UAS*

Forward CCGGCTACTACTACGTGGACTC

Reverse GATTTACTTGGTTGCTGGTTACTTT

*piggyBac* (Left Arm)

Forward (L1) TGTCAATGCGGTAAGTGTCA

Reverse (L2) CCTCTGTGGCAAGGTCAAGA

*piggyBac* (Right Arm)

Forward (R1) TGATGACCTGCAGTAGGAAGACG

Reverse (R2) AGAAACAACTTTGGCACATATCA

*SERCA* (3’UTR)

Forward (R1) TTCTCTTTCTCCTCACGCAGTCAAC

Reverse (R2) GATGGCGCTTTCTTTCACTTACAAT

Gene (qRT-PCR) Sequence 5’-3’

*RPS7*

Forward CCCGGAGCCCTACCTATAAACTAT

Reverse GCAGCACAAAGATGATTTATGCAC

*miR-275*

Forward TCAGGTACCTGAAGTAGCGC

Reverse miRNA Universal primer (Qiagen)

*SERCA*

Forward GATAACCCAATGAAGCCTGATGA

Reverse ACCGACGAAGGTTAGGTTGACT

*NR*

Forward TCGGGCAGAAACTGTGAAATC

Reverse ACTCGGCAGACGCACTTGTAT

*Eubacteria*

Forward TCCTACGGGAGGCAGCAGT

Reverse GGACTACCAGGGTATCTAATCCTGTT

*Flavobacteriaceae*

Forward TAAGGTTGAAGTGGCTGGAATAA

Reverse GTCCATCAGCGTCAGTTAAGACT

*Enterobacteriaceae*

Forward CGTGCTACAATGGCATATACAAAGAGAAG

Reverse AGCATTCTGATCTACGATTACTAGCGATTC

*Acetobacteraceae*

Forward GTGCCGATCTCTAAAAGCCGTCTCA

Reverse TTCGCTCACCGGCTTCGGGT

dsRNA Sequence 5’-3’

*dsLuc*

Forward TAATACGACTCACTATAGGGCTCTGCCTCATAGAACTGCCTG

Reverse TAATACGACTCACTATAGGGAACCTTCGCTTCAAAAAATGGA

*dsSERCA*

Forward TAATACGACTCACTATAGGGAACCCGCCCACAAGTCCAAG

Reverse TAATACGACTCACTATAGGGGGCACCGACATAACCACCAATAG

*dsNR*

Forward TAATACGACTCACTATAGGGTGTAACTGGGACGGTTTGGATTG

Reverse TAATACGACTCACTATAGGGGGATTGACGCCCTTGACTTGATA
